# Physical activity and health-related quality of life after lung cancer surgery– cross-sectional analyses 3 and 12 months postoperatively

**DOI:** 10.1186/s12955-025-02400-z

**Published:** 2025-07-09

**Authors:** Marcus Jonsson, Anita Hurtig-Wennlöf, Anders Ahlsson, Elisabeth Westerdahl

**Affiliations:** 1 Department of Physiotherapy, Faculty of Medicine and Health, Örebro University, Örebro, SE-70182 Sweden; 2 Department of Clinical Diagnostics, School of Health and Welfare, Jönköping University, Jönköping, SE-55111 Sweden; 3 School of Medical Sciences, Faculty of Medicine and Health, Örebro University, Örebro, SE-70182 Sweden; 4 University Health Care Research Center, Faculty of Medicine and Health, Örebro University, Örebro, SE-70182 Sweden

**Keywords:** Thoracic surgery, Postoperative, Health-related quality of life, Lung cancer

## Abstract

**Background:**

Lung cancer is one of the most frequently diagnosed cancers, and the leading cause of cancer deaths worldwide. Surgical resection is the primary curative treatment. The World Health Organization (WHO) recommends that all adults, including cancer survivors, should engage in at least 150 to 300 min of moderate-intensity aerobic physical activity per week. Positive associations have been found between self-reported physical activity and health-related quality of life (HRQoL) after lung cancer surgery. However, there is a lack of studies concerning objectively measured physical activity levels, and longer follow-ups are also missing. This study investigated the relationship between objectively measured physical activity levels and HRQoL in patients 3 and 12 months after lung cancer surgery.

**Methods:**

Utilizing a cross-sectional design, patients were followed up 3 (*n* = 83) and 12 (*n* = 57) months after lung cancer surgery. HRQoL was assessed with the cancer-specific questionnaire EORTC QLQ-C30 and the lung-cancer-specific module LC13. Physical activity was measured with a tri-axial accelerometer (ActiGraph GT3X+).

**Results:**

At 3 months after surgery, 51% (*n* = 42) of the patients reached the level of physical activity recommended by the WHO; the corresponding result at 12 months was 42% (*n* = 24). Patients who reached the recommended level of physical activity reported a better HRQoL, with better global health status and physical function as well as lower symptoms of fatigue, at both 3 and 12 months postoperatively.

**Conclusions:**

Physical activity was positively associated with HRQoL. Encouraging and supporting patients to engage in regular physical activity could contribute to better HRQoL after lung cancer surgery.

**Trial registration:**

The trial was registered at ClinicalTrials.gov (NCT01961700), registration date 20,131,009.

## Background

Lung cancer is one of the most frequently diagnosed cancer, and the leading cause of cancer deaths worldwide [1]. Surgical resection with or without adjuvant chemotherapy is the primary curative treatment of non-small cell lung cancer, and significantly improves mortality rates [2]. The World Health Organization (WHO) recommends that all adults, including those with chronic conditions and those living with disability, should regularly undertake at least 150–300 min of moderate-intensity physical activity per week [3]). An average of 4600 steps/day has been proposed to meet this level of physical activity [4]. However, patients undergoing lung cancer surgery show a low level of physical activity preoperatively [5], and multiple studies have also shown an impaired level of physical activity postoperatively [5–8].

A previous study by Solberg Nes et al. [9] found that the majority of long-term lung cancer survivors reported having a sedentary lifestyle at diagnosis as well as at follow-up. Increased level of self-reported physical activity was associated with improved health-related quality of life (HRQoL), measured with a 10-point linear analog self-assessment scale [9]. Decreased HRQoL has been reported up to 5 years after surgery [10, 11]. Self-reported physical activity has also been positively associated with control of the symptom burden in lung cancer survivors [9]. Patients who are more active in the first month following lung cancer surgery report better health outcome 6 months postoperatively than their less active counterparts [12].

There is a lack of studies investigating repeated objectively measured level of physical activity and its association with HRQoL in a longer perspective after lung cancer surgery. The aim of this study was to investigate the relationship between objectively measured physical activity and self-reported HRQoL in patients 3 and 12 months after lung cancer surgery.

## Methods

### Study design

This study is an exploratory analysis of a previous randomized controlled single-center study that included 107 patients undergoing lung cancer surgery [13]. This analysis utilizes a cross-sectional design. The original trial was registered at ClinicalTrials.gov (https://clinicaltrials.gov/show/NCT01961700), registration date 2013-10-09. The Regional Ethical Review Board in Uppsala, Sweden, approved the study (Ref 2013/199).

### Setting

All patients underwent elective thoracic surgery for suspected or confirmed lung cancer in one university hospital between December 2013 and January 2017. The surgery was performed either by video-assisted thoracoscopy or by open antero-lateral muscle-sparing thoracotomy, according to the surgeon’s preference.

### Participants

Details of the eligibility criteria, sources and methods of participant selection, and methods of follow-up in the primary study have been published previously [13]. In this exploratory analysis, patients with data on physical activity and HRQoL at the two postoperative time-points (3 and 12 months after surgery) were included. The patients were analyzed with regard to whether or not they reached the level of physical activity recommended by WHO [4].

### Variables

Measurements of physical activity and HRQoL were performed 3 and 12 months after surgery.

#### Physical activity

Objective measurement of physical activity was performed using a tri-axial accelerometer (ActiGraph model GT3X+, Manufacturing Technology Inc., Pensacola, FL, USA). The patients were instructed to wear the device on the waist at all times, but were allowed to remove it while sleeping, showering, bathing or swimming. Measurements were performed over 1 week at 3 and 12 months after surgery. Wear time validation was conducted according to Choi [14]. Minimum wear time per day was set to 600 min, and minimum number of wear days was set to 2 days. A cut-off of an average of 4600 steps/day was used as a limit for reaching the recommended level of physical activity according to the WHO [4]. The patients were divided into two groups on the basis of whether or not they reached the cut-off of 4600 steps/day at the two separate time points. Cut-off points according to Barnett [15] were used to calculate sedentary time (0 − 100 counts), light-intensity physical activity (101 − 1012 counts) and moderate and vigorous-intensity physical activity (MVPA) (≥ 1013 counts). All accelerometer data were processed using the designated software (ActiLife version 6.11.9).

#### Health-related quality of life

HRQoL was assessed using the European Organization for the Research and Treatment of Cancer Questionnaire (EORTC QLQ-C30) with the supplemental lung cancer module (LC13). The patients completed the questionnaire at 3 and 12 months after surgery.

The EORTC QLQ-C30 is a standardized, self-administered disease-specific HRQoL instrument designed to measure HRQoL among oncological patients. The supplementary module LC13 consists of 13 questions designed specifically for lung cancer patients. The C30 is composed of nine multi-item scales including five functional scales, three symptom scales, a global quality-of-life scale, and six single-item symptom measures. The LC13 contains one multi-item and nine single-item symptom scales assessing both the symptoms of cancer and the adverse effects of treatment. All items are scored on a scale from 0 to 100. Higher scores on the functional and global quality-of-life scales indicate higher functioning or quality of life, while higher scores on the symptom scales indicate a greater level of symptoms.

### Statistical methods

Patient characteristics were summarized as follows: continuous data as mean ± standard deviation (SD) or median with interquartile range (IQR), and categorical data as numbers and percentages.

To adjust for age differences between patients who reached the physical activity cut-off 3 months after surgery and those who did not, the Quade rank analysis of covariance was employed. Predictors included age, BMI, length of surgery, and length of stay. P-values from the Quade test are reported for differences in HRQoL between the physical activity cut-off groups at 3 months post-surgery.

Differences in HRQoL between the groups 12months after surgery were assessed using the Mann-Whitney U test.

Associations between physical activity and HRQoL were determined using Spearman’s correlation coefficient, interpreted follows: 1.0 perfect, 0.8–0.9 very strong, 0.6 − 0.7 moderate, 0.3 − 0.5 fair, 0.1 poor, 0.0 none [16].

A p-value < 0.05 was considered statistically significant for all analyses. Statistical computations were conducted using version 29 of IBM SPSS Statistics (IBM Corporation, Armonk, New York, USA).

## Results

Data on both physical activity and HRQoL were provided by 83 patients at 3 months after surgery and 57 patients at 12 months after surgery. Descriptive data for the patients at the two time points are presented in Table 1.

**Table 1:** Descriptive data for patients 3 and 12 months after lung cancer surgery

	Patients at 3 months after surgery (*n* = 83)	Patients at 12 months after surgery (*n* = 57)
Age at time of surgery (years)	68 ± 8	69 ± 8
Gender (male/female, %)	43/57	42/58
BMI at time of surgery (kg/m^2^)	26 ± 4	26 ± 4
Smoking history (never/former/current, %)	23/67/10	23/68/9
COPD (yes (%))	8 (10%)	6 (11%)
Type of surgery (thoracotomy/VATS, n)	63/20	45/12
Length of surgery (minutes)	127 ± 52	123 ± 48
Length of hospital stay (days)	4 (3 − 6)	4 (3 − 6)
Sedentary time (minutes/day)	797 ± 201	837 ± 209
Light-intensity physical activity (minutes/day)	125 ± 40	123 ± 43
MVPA (minutes/day)	53 ± 34	53 ± 35
Steps per day	4834 ± 2729	4642 ± 2860

Data presented as mean ± standard deviation, frequency, or median (interquartile range). BMI: body mass index. COPD: chronic obstructive pulmonary disease. MVPA: moderate and vigorous intensity physical activity. VATS: video-assisted thoracoscopic surgery

### Three months after surgery

At 3 months after surgery, 51% (*n* = 42) of the 83 patients reached the level of physical activity recommended by the WHO. There were no significant differences between the patients who did and did not achieve 4600 steps/day in terms of gender, BMI, length of surgery, or length of stay. However, they differed in age, with the less active patients being older than their more active counterparts (mean age 70 ± 7 vs. 66 ± 8, *p* = 0.02). This variation was duly addressed in the statistical analysis, as outlined in the statistical methods section.

Patients who reached the recommended level of physical activity 3 months after surgery reported significantly better global health status, physical function, and role function according to EORTC QLQ-C30 (Table 2). Symptoms of fatigue and appetite loss were also significantly lower in these patients (Table 2).

**Table 2:** Health-related quality of life in patients 3 months after lung cancer surgery

	Patients reaching 4600 steps/day (*n* = 42)	Patients not reaching 4600 steps/day (*n* = 41)	*p*-value
**EORTC QLQ-C30**			
Global health status	67 (50 − 83)	58 (33 − 75)	0.006
Physical function	87 (72 − 93)	67 (47 − 87)	< 0.001
Role function	67 (50 − 83)	67 (33 − 75)	0.043
Emotional function	83 (73 − 93)	92 (47 − 87)	0.727
Cognitive function	100 (83 − 100)	83 (67 − 100)	0.236
Social function	83 (67 − 100)	67 (67 − 100)	0.101
Fatigue	28 (11 − 44)	44 (22 − 61)	0.018
Nausea/vomiting	0 (0 − 0)	0 (0 − 17)	0.062
Pain	17 (0 − 33)	17 (0 − 50)	0.292
Dyspnea	33 (33 − 67)	33 (33 − 67)	0.299
Insomnia	33 (0 − 33)	33 (0 − 67)	0.644
Appetite loss	0 (0 − 0)	0 (0 − 33)	0.025
Constipation	0 (0 − 33)	0 (0 − 0)	0.393
Diarrhea	0 (0 − 0)	0 (0 − 33)	0.673
Financial problems	0 (0 − 0)	0 (0 − 0)	0.685
**LC 13**			
Dyspnea	22 (22 − 33)	22 (22 − 56)	0.127
Coughing	33 (0 − 33)	33 (0 − 67)	0.094
Hemoptysis	0 (0 − 0)	0 (0 − 0)	0.496
Sore mouth	0 (0 − 0)	0 (0 − 0)	0.682
Dysphagia	0 (0 − 0)	0 (0 − 0)	0.351
Peripheral neuropathy	0 (0 − 0)	0 (0 − 33)	0.057
Alopecia	0 (0 − 0)	0 (0 − 0)	0.908
Pain in chest	0 (0 − 33)	0 (0 − 33)	0.541
Pain in arm	0 (0 − 33)	0 (0 − 33)	0.989
Other pain	17 (0 − 67)	33 (0 − 67)	0.193

Health-related quality of life measured with the European Organization for the Research and Treatment of Cancer Questionnaire (EORTC QLQ-C30) and its supplemental lung cancer module (LC13). Data are presented as median (interquartile range). For global health status and the function variables, a higher value represents better health, while for symptom scales and financial problems, a higher score represents worse health

Three months after surgery, there was a fair positive correlation between time spent in MVPA and both global health status (*r* = 0.312, *p* = 0.004) and physical function (*r* = 0.404, *p* < 0.001) (Supplementary Table 1). There were also fair correlations between steps per day and both global health status and physical function (Table 3). Similar correlations were found between time spent in light-intensity physical activity and both global health status and physical function, ranging from poor to fair (Table 3). No correlations were found between sedentary time and any HRQoL domain (Table 3).

**Table 3:** Correlations between HRQoL and objectively measured physical activity (*n* = 83) 3 months after lung cancer surgery

Variable	Sedentary time (average minutes/day)	Light intensity physical activity (average minutes/day)	MVPA (average minutes/day)	Steps per day
Global health status	-0.124	0.237*	0.312*	0.311*
Physical function	-0.001	0.328*	0.404*	0.471*
Role function	0.011	0.234*	0.155	0.201
Emotional function	0.163	0.129	0.010	0.034
Cognitive function	-0.081	0.182	0.168	0.163
Social function	0.020	0.185	0.139	0.213
Fatigue	0.049	-0.222*	-0.213	-0.295*
Nausea/vomiting	-0.008	-0.219*	-0.198	-0.208
Pain	0.078	-0.136	-0.146	-0.106
Dyspnea	0.156	-0.138	-0.023	-0.103
Insomnia	-0.036	-0.087	0.013	-0.013
Appetite loss	0.094	-0.308*	-0.186	-0.202
Constipation	-0.026	0.072	0.040	-0.057
Diarrhea	-0.063	-0.032	0.103	0.013
Financial problems	-0.004	-0.142	0.117	0.126
LC dyspnea	0.166	-0.289*	-0.130	-0.156
LC coughing	-0.057	-0.123	0.024	-0.089
LC haemoptysis	n/a	n/a	n/a	n/a
LC sore mouth	0.090	-0.080	0.066	0.122
LC dysphagia	-0.129	-0.054	0.134	0.135
LC peripheral neuropathy	0.020	-0.186	-0.139	-0.174
LC alopecia	0.000	-0.013	0.026	-0.067
LC pain in chest	0.134	-0.179	-0.088	-0.105
LC pain in arm	0.024	-0.128	-0.043	-0.002
LC other pain	0.063	-0.091	-0.168	-0.183

Health-related quality of life measured with the European Organization for the Research and Treatment of Cancer Questionnaire (EORTC QLQ-C30) and its supplementary lung cancer module (LC13). Physical activity measured with accelerometry. For global health status and the function variables, a positive correlation represents better function, while for symptom scales and financial problems, a negative correlation represents less problem. Correlations presented as Spearman’s rho (p-value). Interpretation of correlation coefficients: 1.0 perfect, 0.8 − 0.9 very strong, 0.6 − 0.7 moderate, 0.3 − 0.5 fair, 0.1 poor, 0.0 none. * indicates statistical significance (*p* < 0.05)

### Twelve months after surgery

At 12 months after surgery, 42% (*n* = 24) of the 57 patients reached the recommended level of physical activity. The proportion of patients reaching or not reaching the recommended level was similar at both 3 and 12 months (*p* = 0.44). There were no statistically significant differences in gender, age, BMI, length of surgery or length of hospital stay between patients who did and did not achieve the recommended level.

The patients who reached the recommended level of physical activity 12 months after surgery reported significantly better global health status and physical function, and significantly lower fatigue scores (Table 4).

**Table 4:** Health-related quality of life in patients 12 months after lung cancer surgery

	Patients reaching 4600 steps/day (*n* = 24)	Patients not reaching 4600 steps/day (*n* = 33)	*p*-value
**EORTC QLQ C30**			
Global health status	83 (67 − 96)	58 (50 − 83)	0.005
Physical function	93 (80 − 100)	60 (40 − 80)	< 0.001
Role function	100 (75 − 100)	50 (33 − 83)	< 0.001
Emotional function	96 (75 − 100)	83 (67 − 100)	0.123
Cognitive function	100 (83 − 100)	83 (67 − 100)	0.105
Social function	100 (100 − 100)	67 (33 − 100)	< 0.001
Fatigue	22 (0 − 33)	44 (11 − 67)	0.004
Nausea/vomiting	0 (0 − 0)	0 (0 − 8)	0.230
Pain	0 (0 − 25)	17 (0 − 50)	0.040
Dyspnea	33 (0 − 33)	33 (33 − 67)	0.041
Insomnia	33 (0 − 33)	0 (0 − 67)	0.699
Appetite loss	0 (0 − 0)	0 (0 − 0)	0.341
Constipation	0 (0 − 33)	0 (0 − 33)	0.564
Diarrhea	0 (0 − 0)	0 (0 − 33)	0.111
Financial problems	0 (0 − 0)	0 (0 − 0)	0.189
**LC 13**			
Dyspnea	17 (11 − 22)	22 (11 − 56)	0.012
Coughing	33 (0 − 33)	33 (0 − 33)	0.924
Hemoptysis	0 (0 − 0)	0 (0 − 0)	0.820
Sore mouth	0 (0 − 0)	0 (0 − 0)	0.754
Dysphagia	0 (0 − 0)	0 (0 − 0)	0.372
Peripheral neuropathy	0 (0 − 33)	0 (0 − 33)	0.328
Alopecia	0 (0 − 17)	0 (0 − 0)	0.546
Pain in chest	0 (0 − 0)	0 (0 − 0)	0.323
Pain in arm	0 (0 − 33)	0 (0 − 33)	0.786
Other pain	0 (0 − 50)	33 (0 − 67)	0.230

Health-related quality of life measured with the European Organization for the Research and Treatment of Cancer Questionnaire (EORTC QLQ-C30) and its supplementary lung cancer module (LC13). Data are presented as median (interquartile range). For global health status and the function variables, a higher value represents better health, while for symptom scales and financial problems, a higher score represents worse health

There were positive correlations between all physical activity variables measured and global health status, physical function, role function, social function, and dyspnea. The correlations ranged from fair to moderate, with the strongest correlations seen between physical function and MVPA (*r* = 0.650) and physical function and steps/day (*r* = 0.687) (Table 5).

**Table 5:** Correlations between HRQoL and objectively measured physical activity (*n* = 57) 12 months after lung cancer surgery

Variable	Sedentary time (average minutes/day)	Light intensity physical activity (average minutes/day)	MVPA (average minutes/day)	Steps per day
Global health status	0.102	0.368*	0.359*	0.394*
Physical function	-0.050	0.424*	0.650*	0.687*
Role function	0.038	0.418*	0.544*	0.598*
Emotional function	0.175	0.147	0.133	0.128
Cognitive function	-0.149	0.184	0.227	0.215
Social function	0.116	0.373*	0.451*	0.502*
Fatigue	-0.049	-0.358*	-0.404*	-0.445*
Nausea/vomiting	-0.051	-0.061	-0.057	-0.084
Pain	-0.065	-0.113	-0.223	-0.267*
Dyspnea	0.005	-0.339*	-0.290*	-0.320*
Insomnia	0.017	-0.042	0.039	0.008
Appetite loss	0.119	0.227	-0.178	-0.218
Constipation	-0.309*	0.105	0.004	0.051
Diarrhea	-0.207	0.046	0.024	-0.018
Financial problems	0.046	-0.223	-0.085	-0.104
LC dyspnea	-0.037	-0.301*	-0.359*	-0.362*
LC coughing	-0.022	0.189	0.101	0.049
LC haemoptysis	0.110	-0.052	-0.035	-0.029
LC sore mouth	-0.117	-0.012	0.084	0.045
LC dysphagia	-0.099	0.057	0.132	0.157
LC peripheral neuropathy	-0.101	-0.119	-0.175	-0.133
LC alopecia	-0.249	0.214	0.044	0.092
LC pain in chest	-0.031	-0.072	-0.089	-0.125
LC pain in arm	-0.011	0.215	-0.094	0.005
LC other pain	-0.071	0.086	-0.112	-0.130

Health-related quality of life measured with the European Organization for the Research and Treatment of Cancer Questionnaire (EORTC QLQ-C30) and its supplementary lung cancer module (LC13). Physical activity measured with accelerometry. For global health status and the function variables, a positive correlation represents better function, while for symptom scales and financial problems, a negative correlation represents less problem. Correlations presented as Spearman’s rho (p-value). Interpretation of correlation coefficients: 1.0 perfect, 0.8 − 0.9 very strong, 0.6 − 0.7 moderate, 0.3 − 0.5 fair, 0.1 poor, 0.0 none. * indicates statistical significance (*p* < 0.05)

## Discussion

This cross-sectional study found a positive relationship between level of physical activity and disease-specific HRQoL; patients reaching the recommended level of physical activity reported significantly better global health status and physical function, both at 3 months and at 12 months after lung cancer surgery.

At 3 months after surgery, 51% of the patients achieved the recommended level of physical activity, and those who did so reported significantly better global health status, physical function, and role function. They also experienced less fatigue and appetite loss compared to their less active counterparts. At 12 months post-surgery, 42% of the patients met the physical activity recommendations. These patients also reported better global health status and physical function, and experienced lower levels of fatigue compared to their less active counterparts. The reported HRQoL values in these components were comparable to those of the Swedish general population for patients reaching the recommended level of physical activity [17]. In contrast, the patients who did not reach the recommended levels showed lower scores in functioning scales and higher symptoms of fatigue than the general population [17].

It has previously been shown that newly diagnosed lung cancer patients with higher levels of physical activity report better HRQoL than those with lower levels of physical activity [18]. Similarly to our results, a study by Zhang et al. found that patients undergoing lung cancer surgery reported impaired HRQoL 2 months postoperatively [19]. Also in line with our results, Pompili et al. [20] showed decreased HRQoL 3 months after lung cancer surgery, with clinically meaningful effects confirmed in social function, physical function, role function, and dyspnea. When comparing results between studies, it is important to be aware of the fact that the characteristics of patients with lung cancer differ depending on region (i.e. Asia versus Europe), ta a large extend related to smoking exposure [1].

The patients in the present study with higher levels of physical activity reported significantly lower symptoms of fatigue at both 3 and 12 months postoperatively. In contrast, D’Silva et al. demonstrated only small associations between MVPA and fatigue, and no associations between MVPA and HRQoL or physical and functional well-being [21]. Their results should, however, be regarded in the light of a response rate of only 24%. When interpreting the results of the present study, the lack of information on disease progress should be considered. It could be argued that patients who are cured would probably experience fewer symptoms and be able to maintain a higher level of physical activity than patients still undergoing adjuvant treatments. Unfortunately, information on adjuvant therapies and disease recurrence was not included in the study design.

Zhou et al. concluded that levels of physical activity are low after lung cancer surgery, and may not recover within 3 months following surgery [5]. In our study, 51% and 42% of the patients reached the recommended level of physical activity at 3 and 12 months, respectively, after surgery. A lower level of physical activity at diagnosis may be a risk factor for HRQoL impairment during and after lung cancer treatment [22]. Being physically active is therefore beneficial for patients with lung cancer. Moreover, engaging in leisure-time physical activity has been associated with improved HRQoL [23].

The findings of the present study underscore the importance of promoting physical activity in a longer perspective as part of postoperative care. Patients achieving the recommended levels of physical activity reported significantly better HRQoL than less physically active patients, suggesting increasing physical activity could be beneficial for patients undergoing lung cancer surgery. Healthcare providers should consider integrating structured physical activity programs and providing resources to support patients in meeting physical activity recommendations. The optimal timing of these interventions remains unclear. The significant association between physical activity and HRQoL observed 3 months after surgery in the present study could support potential benefits of early interventions. Future research should explore the mechanisms underlying the relationship between physical activity and HRQoL, in order to develop targeted rehabilitation for patients undergoing lung cancer surgery.

### Strengths and limitations

The strengths of this study include the use of accelerometry, providing objective and reliable data on physical activity. Another strength is the use of a validated and widely used cancer-specific HRQoL questionnaire, enabling comparability with other studies. Several limitations should be considered when interpreting these results. Firstly, the sample size decreased from 83 participants at 3 months to 57 at 12 months, which may have introduced attrition bias and affected the generalizability of the findings. Information on drop out reason, adjuvant treatment or eventual recurrence was not collected. In line with ethical approval, patients were able to withdraw from the study without providing reason. Secondly, although the variation in age between more and less active groups was accounted for in the statistical analysis, other unmeasured confounding factors could have influenced the outcomes. Thirdly, the observational nature of this study limits the ability to infer causality between higher level of physical activity and better HRQoL. It could be that patients with higher HRQoL or lower symptom burden are able to be more physically active, or that being physically active leads to higher HRQoL and less symptoms. Fourthly, concerns may arise from the methods used to measure physical activity. The use of an accelerometer worn at the waist may not effectively measure some variants of activities, such as riding a bike or activities primarily involving upper body movement. Further, the number of registered days could be regarded as a limitation. Although there is no well-established recommendation, Wendt et al. [24] suggest that 2 or 3 days can be sufficient when measuring physical activity in older individuals, since they present a regular pattern of physical activity throughout the days. When measuring MVPA, 2 days of registration has been recommended [25]. Finally, we did not have any objectively measured data on the patients’ preoperative level of physical activity. Due to the drop-out rate, longitudinal analysis was not applicable.

## Conclusions

In this cohort, half of the patients undergoing lung cancer surgery reached the level of physical activity recommended by the WHO at 3 months after surgery. Patients who reached the recommended level of physical activity reported better global health status and physical function, as well as lower symptoms of fatigue, at both 3 and 12 months postoperatively. Encouraging and supporting patients to engage in regular physical activity could contribute to better HRQoL after lung cancer surgery.

## Data Availability

The datasets used and analyzed during the current study are available from the corresponding author on reasonable request.

## Abbreviations

EORTC QLQ-C30: European Organisation for Research and Treatment of Cancer Quality of Life Questionnaire Core 30
LC13: EORTC QLQ-C30 lung cancer module
MVPA: Moderate and vigorous-intensity physical activity
WHO: World Health Organization
